# Adapting a resistance training program for Danish physical education: teachers' preconceptions and participatory co-adaptation

**DOI:** 10.3389/fspor.2026.1816683

**Published:** 2026-04-15

**Authors:** Sofie Koch, Marianne Friis Andersen, Malte Nejst Larsen, Jesper Ninn Sandfeld Melcher, Thomas Viskum Gjelstrup Bredahl, Christina Birch Meiner-Jensen, David R. Lubans, Lars Breum Christiansen

**Affiliations:** 1 Research Unit for Active Living, Department of Sports Science and Clinical Biomechanics, University of Southern Denmark, Odense, Denmark; 2 Research and Implementation Centre for Human Movement and Learning, Department of Sports Science and Clinical Biomechanics, University of Southern Denmark, Odense, Denmark; 3 Research Unit for Sport and Health Sciences, Department of Sports Science and Clinical Biomechanics, University of Southern Denmark, Odense, Denmark; 4 Faculty of Teacher Education, University College Copenhagen, Copenhagen, Denmark; 5 Danish Centre for Motivation and Behaviour Science (DRIVEN), Department of Sports Science and Clinical Biomechanics, University of Southern Denmark, Odense, Denmark; 6 Global Sport and Movement Collaborative, University of Newcastle, Newcastle, NSW, Australia; 7 Hunter Medical Research Institute, New Lambton, NSW, Australia

**Keywords:** adolescents, cultural adaptation, implementation, physical education, scaling

## Abstract

**Introduction:**

Transferring and scaling school-based physical activity (PA) programs across educational contexts requires systematic adaptation to ensure implementation feasibility and contextual fit. This study examined Danish physical education (PE) teachers' preconceptions for resistance training and a participatory co-adaptation process used to adapt the evidence-based Resistance Training for Teens (RT4T) program for implementation in Danish lower secondary schools.

**Methods and materials:**

MEGAFiT, a Danish adaptation of RT4T, was developed using a sequential qualitative design. We conducted six online group interviews with 19 PE teachers and analysed data using the COM-B model to identify key implementation determinants related to capability, opportunity, and motivation. These findings informed four participatory co-adaptation workshops with 14 PE teachers, combining practical testing, reflection, and iterative program refinement. The resulting MEGAFiT program was structured as a six-week PE course consisting of modular, equipment-light resistance training sessions designed to integrate with PE content. Adaptations were documented using the Framework for Reporting Adaptations and Modifications-Expanded (FRAME).

**Results:**

Teachers' capability and confidence to deliver resistance training varied substantially and were closely linked to prior experience. Key implementation barriers included limited lesson time, large class size, and restricted access to facilities and equipment. During the co-adaptation process, teachers and researchers collaboratively refined the program by shortening its duration, introducing modular lesson structures, developing equipment-light exercise formats, and redesigning instructional materials to improve usability in PE settings.

**Conclusion:**

Explicitly addressing implementer preconceptions through structured co-adaptation strengthened the alignment between the RT4T program and the Danish school context. The findings illustrate how participatory adaptation can help tailor evidence-based PA programs to everyday PE practice by aligning program structure, organization, and materials with teachers' capabilities and contextual constraints.

## Introduction

Physical inactivity among adolescents remains a persistent global health concern and is associated with increased risks of physical, cognitive, and mental health problems across the life course (1). Globally, more than 80% of adolescents do not meet the World Health Organization (WHO) recommendation of at least 60 min of daily moderate-to-vigorous intensity physical activity (MVPA) (2). In Denmark, only 26% of adolescents are sufficiently active (3). The WHO guidelines further emphasize the importance of including muscle- and bone-strengthening activities at least three times per week to support healthy growth, bone density, and muscular development. While such activities can be integrated into the daily MVPA recommendation, their implementation requires age-appropriate progression and pedagogical adaptation, particularly in school settings (4).

A growing body of evidence demonstrates that appropriately designed resistance training is safe and beneficial for children and adolescents, with documented effects on muscular fitness, body composition, injury prevention, mental health, and physical literacy (5–8). Resistance-based activities may also appeal to adolescents who are less engaged by traditional team sports, thereby offering a potentially inclusive approach to PA promotion. From a health promotion perspective, schools represent a key setting for delivering such activities, as they provide structured opportunities to reach adolescents across social groups (9). Despite this, resistance training remains underrepresented in school-based physical education (PE), particularly in lower secondary education (10, 11).

From an implementation perspective, limited uptake of resistance training in PE cannot be explained by lack of evidence alone. Previous research highlights several implementation challenges, including teachers' limited formal training in resistance training exercise pedagogy, safety concerns, time constraints, large class sizes, and restricted access to facilities and equipment (6, 11). These barriers underscore the importance of understanding teachers' implementation preconceptions—such as perceived capability, opportunity, and motivation—as key determinants of whether and how evidence-based programs are adopted, adapted, and sustained in school contexts (12). Such preconceptions may also shape how implementers engage in adaptation processes when interventions are transferred to new educational contexts.

One evidence-based program designed to address adolescents' resistance training competence is *Resistance Training for Teens* (RT4T). Developed and tested across diverse Australian secondary school settings, RT4T has demonstrated positive effects on students' muscular fitness, movement competence, and confidence (7). The program is grounded in the SAAFE pedagogical principles—Supportive, Active, Autonomous, Fai, and Enjoyable—which aim to foster a motivating and psychologically safe learning environment (13). RT4T is intentionally structured to be scalable, using modular formats, minimal equipment, and clear progression principles. However, transferring such programs across cultural and educational contexts requires careful adaptation to local pedagogical traditions, organizational structure, and teacher capabilities to ensure contextual fit between the intervention design and everyday educational practice.

An initial Danish feasibility study demonstrated that a translated version of the RT4T program was acceptable and feasible in Danish secondary schools, while also identifying ongoing challenges related to teacher confidence, curricular alignment, and organizational constraints (14). These findings highlight that successful implementation depends not only on program design, but also on how well interventions align with teachers' everyday practices and contextual conditions. Implementation research increasingly emphasizes that such alignment is best achieved through structured, participatory adaptation processes that actively involve implementers in tailoring intervention components while preserving core program principles and addressing implementers' existing preconceptions regarding feasibility and delivery (15, 16).

Co-adaptation approaches—where researchers and practitioners collaboratively refine program content, structure, and delivery—are increasingly recognized as implementation strategies that can enhance contextual fit, acceptability, and feasibility, while simultaneously supporting professional learning and ownership among implementers (16, 17). Within school-based health promotion, such approaches have been highlighted as particularly relevant for addressing contextual constraints that shape everyday delivery of interventions. However, empirical research remains limited on how teachers' implementation preconceptions shape co-adaptation processes during early implementation phases, particularly when evidence-based programs are transferred across educational and cultural contexts. While previous feasibility studies have primarily examined program acceptability and initial implementation, less is known about how teachers' implementation preconceptions actively inform adaptation decisions during early implementation planning.

Building on prior feasibility work (14) and situated within a school-based health promotion context, the present study examines Danish physical education (PE) teachers' implementation preconceptions related to integrating resistance training into PE and explores how these preconceptions informed a structured participatory co-adaptation process to adapt the evidence-based Resistance Training for Teens (RT4T) program for Danish lower secondary schools. By examining both teachers' implementation preconceptions and the resulting program adaptation, the study provides empirical insight into how participatory co-adaptation can support contextual fit between intervention design and everyday PE practice during early phases of implementation and adaptation.

To guide the study, two research questions were addressed:

1. What implementation preconceptions do Danish PE teachers hold regarding the integration of resistance training in PE?
2. How do these preconceptions inform the participatory co-adaptation of the RT4T program for implementation in Danish lower secondary schools?

## Methods

### Study design

A sequential qualitative design was employed, combining semi-structured group interviews with participatory co-adaptation workshops. Findings from the interviews informed the design and focus of the subsequent co-adaptation workshops (18). The study was conducted as a collaborative partnership between the University of Southern Denmark, Danish School Sport, and University College Copenhagen, integrating expertise, practitioner-oriented facilitation, and educational practice to support the adaptation process.

Multiple implementation frameworks were used with distinct and complementary roles in different phases of the study. The COM-B model guided the development of the interview guide and the analysis of teachers' implementation preconceptions related to capability, opportunity, and motivation (19). The PRACTIS guide informed the structuring of the co-adaptation process with particular attention to feasibility and potential scalability within school settings (20). The Framework for Reporting Adaptations and Modifications-Expanded (FRAME) was used to systematically document the nature, rationale, and contextual drivers of program adaptation identified during the workshops (21). The frameworks were applied pragmatically to support applied inquiry rather than prescriptively as rigid analytical templates. In addition, the SAAFE pedagogical principles underpinning the original RT4T program were treated as core pedagogical components and were maintained throughout the co-adaptation process.

### Study population and recruitment

Between October and November 2024, eight lower secondary schools were recruited through direct outreach, LinkedIn announcements, and dissemination via the Danish School Sport network. Schools were purposively selected to ensure geographic representation across Denmark, with four schools located in the eastern part Denmark and four in the western part of Denmark. This distribution also ensured sufficient local participation to facilitate interactive co-adaptation workshops in both regions.

Nineteen PE teachers participated in the group interviews. Four of these teachers did not participate in the subsequent co-adaptation workshops. In addition, one teacher participated in the workshops without having taken part in the initial interview but was employed at a school that had participated in the interviews. In total, 15 teachers therefore participated in both phases of the study. The reduced participation in the workshops primarily reflected constraints related to teachers' available time and school resources rather than withdrawal from the study.

All participants were practicing PE teachers teaching students in grades 7–9 (approximately 12–16 years). Teachers were recruited based on their interest in participating in the development and adaptation of a resistance training program for PE. While participants shared a general interest in fitness and resistance training, they varied in the extent to which they had previously integrated such activities into their PE teaching.

### Data collection

#### Group interviews

Six semi-structured group interviews were conducted online in December 2024 with 19 PE teachers (two to four per interview) using Microsoft Teams. Interviews lasted between 58 and 84 min (mean: 71.5 min). Small group sizes (two to four participants per interview) were used to facilitate active participation and allow teachers to elaborate on their experiences while still enabling interaction among participants.

The group interview format was chosen to elicit individual perspectives within a shared setting without aiming for consensus. While group discussions may introduce potential influences such as social desirability or professional self-presentation, participants were encouraged to share both positive and critical reflections on implementing resistance training in PE.

The interviews were moderated by the second author, who has experience in conducting qualitative research and in PE contexts. The moderator followed a semi-structured interview guide and encouraged all participants to contribute to the discussion. To minimize researcher influence, the moderator adopted a facilitative role, focusing on open-ended questions and allowing participants to discuss and elaborate on each other's perspectives.

An interview guide structured around the COM-B domains—capability, opportunity, and motivation—was used to explore teachers' experiences, perceived barriers, and facilitators related to implementing resistance training in PE as part of everyday practice. All interviews were audio-recorded and transcribed verbatim. Interviews were conducted with teachers from all participating schools. While the number of interviews was determined by recruitment rather than saturation criteria, preliminary analysis indicated that no substantially new implementation-related themes emerged after the first three to four interviews.

#### Co-adaptation workshops and observations

Four co-adaptation workshops were conducted between March and May 2025 with 16 PE teachers from six schools. Of these, 15 had previously taken part in the group interviews, while one additional teacher joined the workshop phase from one of the schools that had participated in the interviews. The workshops aimed to collaboratively adapt and contextualize the RT4T program for the Danish school context and to facilitate structured reflection on teachers' implementation preconceptions and potential adaptations to program structure, activities, and teaching materials. Each workshop lasted approximately 4.5 h and was facilitated by representatives from Danish School Sport. Members of the research team contributed by providing input related to the research objectives and by observing teachers' reflections and emerging adaptation suggestions during the sessions.

The workshop combined practical testing of selected exercises and teaching activities with group discussions focusing on feasibility, pedagogical alignment with PE curricula, and potential modifications to support implementation in everyday school settings. Teachers were encouraged to reflect on how the program activities aligned with their existing teaching practices and to identify practical barriers and opportunities for integrating resistance training into PE lessons.

The workshops followed a structured and iterative format combining introduction to core RT4T principles and materials, hands-on testing of activities and lesson formats, collective reflection on feasibility, safety, and pedagogical alignment, and refinement of program components based on teachers' feedback. This format was designed to support reflection on how resistance training could be delivered under real-world school conditions rather than idealized training environments.

Between workshops, teachers pilot-tested selected program components in their own PE classes, initially from RT4T and subsequently from the emerging MEGAFiT program. These pilot tests provided practical insights into feasibility, student engagement, and organizational fit, which were discussed and incorporated into subsequent workshops. Workshops were scheduled approximately two weeks apart to follow time for classroom testing and reflection.

Systematic observations were conducted during all co-adaptation workshops and subsequent classroom pilot testing. Observations were carried out by members of the research team and documented as field notes using a structured template adapted from the FRAME framework. The observations focused on documenting the nature and rationale of emerging adaptations, the contextual conditions prompting modifications, and how fidelity to core program principles was maintained alongside flexibility. Observation notes captured teachers' discussions, reflections, and decision-making processes throughout the co-adaptation workshop, with particular attention to contextual constraints shaping feasible delivery. These observational data complemented the interview data and informed the ongoing refinement of the MEGAFiT program as well as the systematic documentation of program adaptations.

### Ethical considerations

The study was approved by the Regional Research Ethics Committee (Protocol no. REC609). All participants provided informed consent and were informed of their right to withdraw at any time without consequence. Data were anonymized and stored in accordance with Danish regulations and the EU General Data Protection Regulation (GDPR). The study adhered to the Declaration of Helsinki and was preregistered on the Open Science Framework (OSF) to ensure transparency and reproducibility (https://osf.io/34ajt/overview).

### Data analysis

Interview transcripts and workshop observation notes were imported into NVivo 15 and analyzed using a framework-based thematic approach suited for applied qualitative research (22–24). The analysis was conducted in several steps to ensure systematic and transparent handling of data.

Interview data were first read repeatedly to ensure familiarity with the data before the coding process was initiated. Interview data were first analyzed deductively using the COM-B model as an organizing framework. Data segments were coded within the three overarching domains—capability, opportunity, and motivation—and their respective subcomponents (capability: physical and psychological; opportunity: physical and social; motivation: reflective and automatic). Initial coding focused on identifying statements reflecting teachers' experiences, perceived barriers, and facilitators related to implementing resistance training in PE. Through iterative comparison across interviews, similar codes were grouped into broader categories capturing recurring implementation considerations related to the COM-B domains. These categories were subsequently refined into themes describing how teachers' implementation preconceptions shaped perceptions of feasibility and potential program adaptations. This approach supported systematic identification of implementation-relevant considerations while maintaining focus on applied, practice-oriented insights.

Observational data from the co-adaptation workshops were analyzed separately using categories derived from the FRAME framework. This analysis focused on documenting the nature of program adaptations, the contextual conditions prompting modifications, and the rationale underlying adaptation decisions, with attention to both fidelity to core program principles and necessary flexibility. Observation notes were examined to identify how teachers' implementation preconceptions were discussed and translated into concrete adaptation suggestions during the workshops and pilot testing sessions.

In the final analytic step, findings from the interview and observational analyses were integrated to examine how teachers' identified preconceptions were reflected in, and informed, subsequent program adaptations during the co-adaptation workshops. This integrative approach enabled triangulation of perceived implementation considerations with observed adaptation practices, strengthening the relevance of findings of school-based health promotion and implementation planning.

To enhance analytical rigor, two authors (SK and MFA) independently coded the data and met regularly to discuss coding decisions, framework application, and theme development. Discrepancies were resolved through discussion until consensus was reached. This iterative process supported refinement of code definitions and ensured consistent interpretation of themes across the dataset.

## Results

Findings from the group interviews and observations conducted during the co-adaptation workshops are presented according to the three core components of the COM-B model: Capability, Opportunity, and Motivation. Within each domain, results describe teachers' implementation preconceptions and illustrate how these were reflected in considerations of feasibility and delivery within routine PE practice during the co-adaptation process.

### Capability: variation in competence and need for professional support

Interview data indicated substantial variation in teachers' perceived capability, partly reflecting differences in prior experience with teaching fitness and resistance training in PE. Teachers with prior personal experience in resistance training described feeling confident in demonstrating exercises, explaining technique, and guiding progression. In contrast, other teachers expressed uncertainty regarding correct execution, safety considerations, and progression beyond basic exercises:

“I don't necessarily think I'm skilled enough in resistance training. I probably haven't focused much on that part, so I don't know exactly how to perform all those. … at least if it gets advanced.” (interview 3, participant 9)

Several teachers attributed this uncertainty to limited exposure to resistance training during teacher education, reflecting constraints related to psychological capability. Interviewees emphasized that hands-on experience was a prerequisite for teaching resistance training effectively:

“I don't think you can just go in and put together a training program if you don't have a prior understanding of what it is. And I also don't think you can demonstrate the exercises without having tried them yourself.” (interview 5, participant 14)

Workshop observations supported these findings. During hands-on testing of exercises and lesson formats, teachers frequently requested clarification regarding technique and progression. Observations further showed that collective experimentation and peer discussion functioned as mechanisms for negotiating adaptations that balanced technical correctness with pedagogical feasibility under real-world teaching conditions.

### Opportunity: organizational, physical, and social conditions for implementation

Interview data highlighted limited access to resistance training equipment, time constraints, and large class sizes as key barriers to implementation. Teachers described teaching classes of 20–28 students, and in some cases multiple classes combined, which restricted opportunities for individual supervision and limited the use of equipment:

“If it has to be really, really, really good, then we need more equipment. We need weights and something we can adjust to increase or decrease the load, and we just don't get that. It's too expensive” (interview 1, participant 4)

Time constraints were also emphasized. PE lessons typically consisted of one weekly double lesson (2 × 45 min), which teachers perceived as insufficient for combining instruction, exercise execution, supervision, and progression. Some teachers reported teaching longer PE blocks, which allowed more time for practice but posed challenges related to sustaining student engagement:

“It's a challenge when you have three lessons in a row. That's a long time for PE. So, you are already challenged in keeping the students engaged.” (interview 5, participant 15)

Workshop observations mirrored these constraints. Teacher repeatedly adapted exercises to reduce reliance on equipment and discussed organizational formats that would allow all student to remain active simultaneously, thereby maximizing active time within the fixed duration of a PE lesson. These discussions consistently focused on what was perceived as feasible within everyday school conditions rather than idealized training environments.

Interview data further indicated that social dynamics influenced the feasibility of resistance training in PE. While many teachers described strong student interest, particularly among performance-oriented students, concerns related to peer comparison and bodily exposure were also raised:

“I think it's really important to tell the students: physical training—it's your body, you feel it yourself. No one else can say, “Oh, is that all you can lift?” or “Why aren't you doing more?” So, I hope physical training can also be an eye-opener for some. Especially, those who maybe don't exercise much or are a bit vulnerable” (interview 5, participant 14)

Teachers also emphasized the importance of framing resistance training as an individual process rather than a competition:

“It's the competition with yourself. You shouldn't compare yourself to everyone else. You should compare yourself to yourself and your own progression in the different exercises” (interview 2, participant 6)

Workshop observations illustrated how these social considerations informed organizational practice. Teachers tested station-based formats, small-group organization, and parallel activities to minimize waiting time and spectator roles, which were perceived as potential sources of discomfort and social comparison, particularly for some students. These organizational strategies were discussed as ways of supporting inclusive participation and a safe learning environment within routine PE lessons.

### Motivation: perceived pedagogical value and professional engagement

Interview data suggested that teachers' motivation to integrate resistance training was closely linked to its perceived pedagogical value. Resistance training was described as adaptable and inclusive, allowing students with diverse abilities to participate and experience success:

“I think it's one of those content areas where students don't need to have good ball skills. So, others can shine in it. More students can participate in it than in a handball match, for example. Because you can bring everything down to a basic level.” (interview 3, participant 11)

Teachers highlighted the potential of resistance training to engage students who were less motivated by traditional team sports and to support confidence, body awareness, and enjoyment of PA. These perceived student benefits appeared closely linked to teachers' willingness to invest effort in adapting and integrating resistance training into their existing PE practice.

Workshop observations complemented these findings. During the co-adaptation workshops, high levels of engagement were observed when teachers collaboratively tested exercises, reflected in classroom pilot tests, and exchanged experiences. These interactions were characterized by shared problem-solving and professional dialogue, suggesting that participation in the co-adaptation process supported teachers' confidence and ongoing motivation to work with resistance training in PE.

### Linking implementation preconceptions and program adaptations

Across interviews and workshop observations, teachers' implementation preconceptions related to capability, opportunity, and motivation were consistently reflected in the program adaptations developed during the co-adaptation process. These adaptations addressed both individual-level teaching considerations and contextual constraints shaping feasible delivery within everyday PE practice.

**Figure 1:**
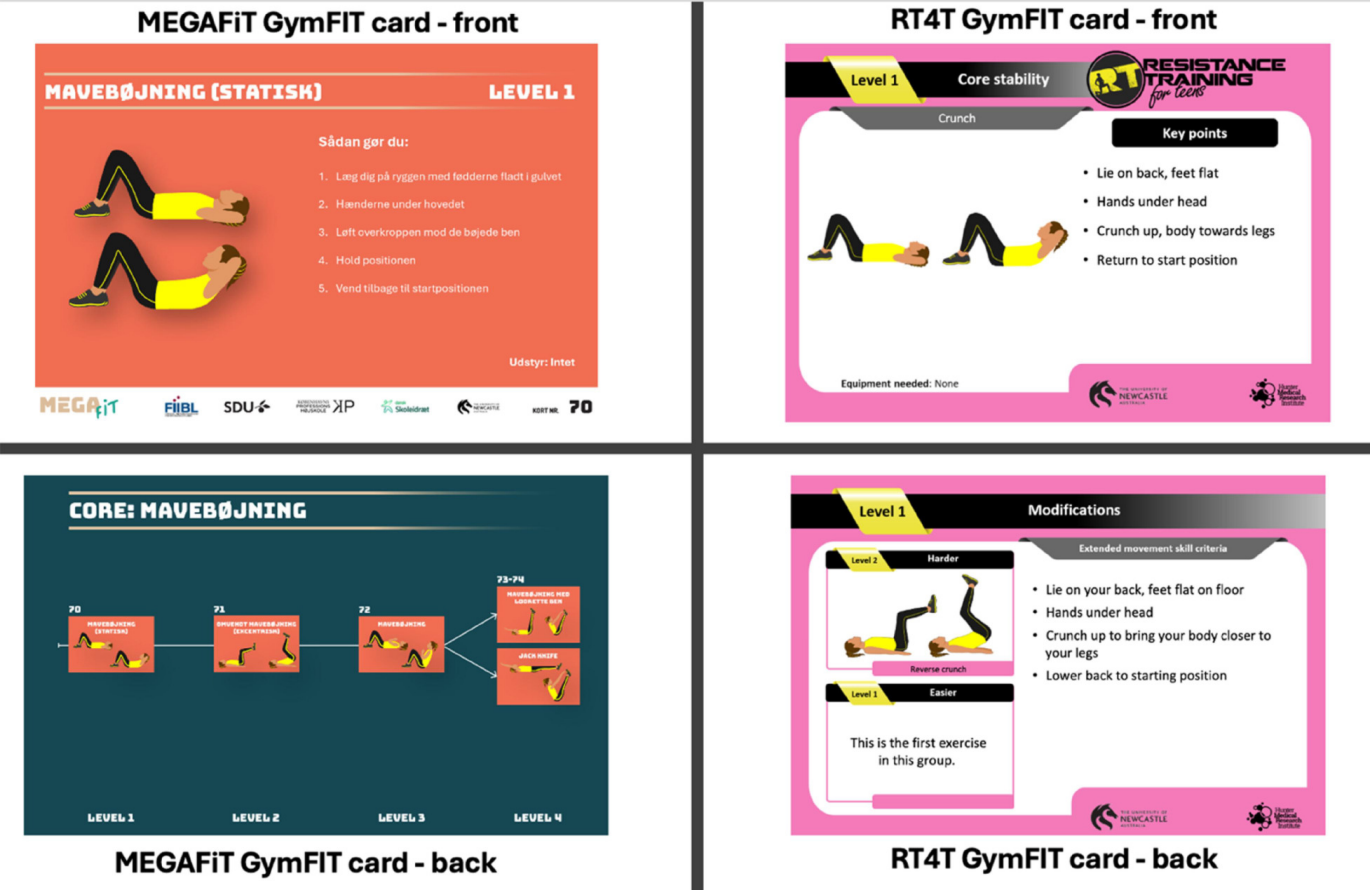
Comparison of original RT4T and adapted MEGAFiT instructional cards highlighting changes in layout and visual integration.

To support teachers' capability, adaptations primarily focused on the presentation and usability of existing program materials rather than changes to core exercise content. Based on teachers' feedback and workshop observations, instructional materials were reorganized so that information about exercises, progression options, and materials needed was visually integrated with corresponding images and illustrations (see Figure 1). This layout aimed to make key information immediately accessible during teaching situations, reducing the need to navigate between separate textual and visual resources. Workshop observations showed that teachers and students frequently relied on visual demonstrations and quick reference points when planning and delivering lessons.

Adaptations targeting physical and organizational opportunity addressed constraints related to time, equipment, and class size. Flexible, modular session structures were developed that required minimal or no specialized equipment and could be scaled to available resources and lesson duration. Organizational formats—such as circuit-based stations, small-group work, and parallel activities—were used to enable simultaneous participation, reduce waiting time, and limit spectator role under typical school conditions.

In addition, the overall structure and duration of the program were adapted to fit teachers' organizational realities and concerns about student engagement. While RT4T was delivered over eight weeks, teachers expressed limited room in annual teaching plans and concerns that a longer program could challenge sustained student motivation. Consequently, the MEGAFiT program was structured as a six-week course. The first four lessons focused explicitly on MEGAFiT content, while the final two lessons emphasized integrating resistance training principles into other PE content areas, such as gymnastics or ball games. This structure was discussed as a way to support transfer of resistance training principles into broader PE practice while maintaining feasibility within existing curricula.

Social opportunity and motivational considerations were addressed through adaptations that emphasized autonomy, self-paced progression, and a supportive learning climate. Exercises and lesson formats were designed to minimize social comparison and performance pressure, while playful and game-based elements were integrated in the beginning and in the end of every lesson to encourage engagement and enjoyment. Teachers also emphasized embedding theoretical content directly within practical PE lessons, resulting in theory being integrated into MEGAFiT sessions rather than delivered as stand-alone lessons, as in the RT4T program. These adaptations reflected teachers' intentions to create inclusive learning environments that supported participation among diverse student groups.

Together, these adaptations illustrate how teachers' identified implementation preconceptions informed the refinement of the MEGAFiT program. By aligning program components with teachers' capabilities, organizational conditions, and pedagogical priorities, the co-adaptation workshops resulted in a program configuration that reflected everyday PE practice and contextual feasibility. In this way, the study illustrates how commonly reported implementation barriers can inform practical program refinements when addressed through participatory co-adaptation processes.

## Discussion

This study examined Danish PE teachers' implementation preconceptions for resistance training and explored how these preconceptions informed the co-adaptation of the RT4T program into the MEGAFiT program for a Danish school context. By explicitly focusing on implementation preconceptions, contextual fit, and implementation feasibility during the implementation phase, the study provides empirical insight into how co-adaptation can support alignment between evidence-based programs and everyday educational practice during early implementation and adaptation processes (15, 20, 21). Integrating interview data with observations from co-adaptation workshops and classroom pilot testing, the findings illustrate how teachers' capabilities, contextual conditions, and pedagogical priorities shape the feasibility of implementing resistance training in routine PE practice. Several of the implementation considerations identified in this study—such as time constraints, equipment limitations, and variation in teacher confidence—have been reported in previous research on school-based PA interventions (25). However, the present study contributes to the literature by illustrating how such implementation preconceptions were translated into concrete program adaptations through a structured co-adaptation process. In this way, the findings demonstrate how teachers' implementation preconceptions informed program refinements that strengthened contextual fit between the intervention design and everyday PE practice. In doing so, the study provides insight into how implementation considerations emerging from teachers' perspectives can inform the adaptation of school-based PA interventions under real-world conditions, corroborating previous research showing that many PE teachers report limited confidence and perceived expertise in delivering resistance or muscular fitness activities (10, 26).

An important feature of the co-adaptation process was the opportunity for teachers to pilot-test program components in their own PE classes between workshops. This pilot testing enabled teachers to assess implementation feasibility under real-world conditions and to reflect on how identified implementation preconceptions manifested in everyday practice. In this way, pilot testing functioned as a practical mechanism for linking intervention principles with routine delivery conditions.

### Teachers' implementation preconceptions as central determinants

Capability-related preconceptions were primarily linked to teachers' confidence in exercise technique, safety, and progression, particularly among those with limited prior experience in resistance training. Previous research has shown that limited confidence and training in resistance exercise can constrain teachers' willingness to implement such activities in school settings (6). The present findings suggest that limited capability does not necessarily prevent implementation but instead shapes the types of support, materials, and organizational formats teachers perceive as feasible within routine PE practice.

Preconceptions related to physical and organizational opportunity—such as limited lesson time, large class sizes, and restricted access to equipment—were consistently highlighted. These factors are widely recognized barriers in school-based PA interventions (12, 25). In the present study, however, they functioned less as isolated barriers and more as contextual conditions shaping teachers' perceptions of feasible delivery within lower secondary PE. Time constraints in particular interacted with class size, equipment availability, and lesson organization to influence how resistance training could be integrated alongside existing curricular demands (25, 27).

Motivational preconceptions were closely aligned with teachers' pedagogical values, particularly the perceived inclusivity and adaptability of resistance training for diverse student groups. These findings align with implementation research emphasizing the importance of congruence between intervention characteristics and implementers' professional beliefs (12, 25). Notably, much of the existing evidence on barriers to school-based PA implementation derives from primary school settings and policy-level interventions (27), with comparatively few studies examining resistance training in secondary school PE. The present study therefore extends previous work by highlighting how these implementation considerations are experienced and negotiated within lower secondary PE contexts.

### Co-adaptation as a bridge between evidence and context

The co-adaptation workshops provided a structured setting in which teachers' implementation preconceptions could be collectively interpreted and translated into concrete program refinements. Rather than identifying barriers in isolation, the workshops enabled teachers and researchers to examine how contextual constraints such as limited time, equipment availability, and class size influenced the feasibility of implementing resistance training into PE practice.

Several of the resulting adaptations reflected efforts to align the program with these contextual conditions, including modifications to exercise structure, equipment use, and instructional materials. Such adaptations illustrate how co-adaptation can support contextual fit by aligning evidence-based program components with the practical realities of school-based implementation. These findings align with implementation research emphasizing that participatory adaptation processes can help balance fidelity to core intervention principles with the flexibility required for real-world delivery (20, 21).

In this way, the study illustrates how teachers' implementation preconceptions can serve as valuable sources of contextual knowledge during early implementation phases, informing program refinements that enhance feasibility and acceptability within everyday PE practice. From an implementation research perspective, this finding supports recommendations to address implementation preconceptions early in implementation planning, while allowing co-adaptation processes to translate these considerations into practical program configurations aligned with local delivery conditions (20, 21).

### Implications for implementation research

This study contributes to implementation research by illustrating how participatory co-adaptation can inform program design and implementation planning without requiring full-scale co-adaptation with all future implementers. The co-adaptation workshops did not constitute a scalable implementation strategy in themselves but generated empirically grounded insights into contextual fit and implementation considerations relevant for future implementation planning. This finding aligns with calls for increased attention to early-phase implementation planning and systematic assessment of implementation preconceptions in complex interventions (20).

Although the present study does not examine large-scale dissemination directly, the findings may provide early-stage insights relevant for future implementation and scaling considerations. Evidence from school-based PA interventions indicates that intervention effectiveness and implementation quality often decrease when programs are scaled up, a phenomenon referred to as voltage drop (28). In their RE-AIM evaluation of the scaled-up RT4T program, Kennedy et al. (28) reported reduced fidelity and attenuated effects compared with earlier trials, underscoring the challenges of maintaining implementation quality during broader dissemination. In this context, early-phase co-adaptation may provide a mechanism for informing intervention design in ways that better reflect delivery conditions prior to large-scale roll-out.

While the specific adaptations identified in this study reflect the Danish PE context, the underlying process of identifying and responding to implementation preconceptions may be transferable to other school-based PA programs operating under similar organizational and pedagogical constraints (15, 21).

### Implications for practice

For educational practice, the findings suggest that successful integration of resistance training in PE may depend less on introducing new content and more on organizing and embedding existing content in ways that enhance contextual fit with teachers' everyday constraints and pedagogical priorities. Flexible program structures, integration across content areas, and attention to organizational and social dynamics may support sustained engagement among both teachers and students, consistent with previous research on sustainable and inclusive PE practice (25, 29).

### Strengths and limitations

Key strengths of this study include the use of multiple qualitative data sources and the integration of interview, observational, and pilot-testing data, which enabled triangulation of perceived implementation preconceptions with observed practice during the co-adaptation process (18, 30). The close collaboration between research and practice-oriented institutions further supported the relevance and feasibility of the adaptation process.

Some limitations should be acknowledged. Participants were self-selected and generally physically active, which may limit transferability to teachers with less interest or experience in resistance training (31). Furthermore, the study focused on the early implementation phase and implementation feasibility and did not examine long-term adoption or sustainability. Future research could explore how the adapted program is implemented over time and how implementation preconceptions evolve during later stages of implementation. Finally, the study focused primarily on teachers' perspectives, capturing their perceived barriers and facilitators to implementation. Broader stakeholder perspectives, such as school leadership, parents, or policy actors, were not included, which may have provided additional insight into organizational and contextual factors influencing program adoption and sustainability (20).

## Conclusions

This study demonstrated that Danish PE teachers' implementation preconceptions related to capability, opportunity, and motivation play an important role in shaping how resistance training programs are adapted for school contexts. Through a structured co-adaptation process supported by classroom pilot testing, these preconceptions informed adaptations to program structure, organization, and usability that strengthened contextual fit without altering core components of the RT4T program.

The findings highlight how participatory co-adaptation can support translation of evidence-based PA programs into educational practice by making explicit the contextual conditions under which they are likely to be delivered. In doing so, the study provides practice-relevant insight into how implementation considerations emerging from teachers' perspectives can inform the adaptation and implementation planning of school-based PA intervention in real-world educational settings.

## Data Availability

The raw data supporting the conclusions of this article will be made available by the authors, without undue reservation.
